# Interpersonal theory of suicide: prospective examination

**DOI:** 10.1192/bjo.2020.93

**Published:** 2020-09-22

**Authors:** Thomas Forkmann, Heide Glaesmer, Laura Paashaus, Dajana Rath, Antje Schönfelder, Katharina Stengler, Georg Juckel, Hans-Jörg Assion, Tobias Teismann

**Affiliations:** Department of Clinical Psychology, University of Duisburg-Essen, Germany; Department of Medical Psychology and Medical Sociology, University of Leipzig, Germany; Department of Clinical Psychology, University of Duisburg-Essen, Germany; Department of Clinical Psychology, University of Duisburg-Essen, Germany; Department of Medical Psychology and Medical Sociology, University of Leipzig, Germany; Department of Psychiatry, Psychosomatic Medicine and Psychotherapy, Helios Park Hospital Leipzig, Germany; Department of Psychiatry, LWL-University Hospital, Ruhr-Universität Bochum, Germany; LWL-Clinic Dortmund, Germany; Mental Health Research and Treatment Center, Department of Psychology, Ruhr-Universität Bochum, Germany

**Keywords:** Suicide attempts, interpersonal theory of suicide, perceived burdensomeness, thwarted belongingness, capability for suicide

## Abstract

**Background:**

The interpersonal theory of suicide (IPTS) is one of the most intensively researched contemporary theories on the development of suicidal ideation and behaviour. However, there is a lack of carefully conducted prospective studies.

**Aims:**

To evaluate the main predictions of the IPTS regarding the importance of perceived burdensomeness, thwarted belongingness and capability for suicide in predicting future suicide attempts in a prospective design.

**Method:**

Psychiatric in-patients (*n* = 308; 53.6% (*n* = 165) female; mean age 36.82 years, s.d. = 14.30, range 18–81) admitted for severe suicidal ideation (*n* = 145, 47.1%) or a suicide attempt completed self-report measures of thwarted belongingness, perceived burdensomeness, capability for suicide, hopelessness, depression and suicidal ideation as well as interviews on suicide intent and suicide attempts and were followed up for 12 months. Logistic regression and receiver operating characteristics (ROC) analysis were conducted.

**Results:**

The interaction of perceived burdensomeness, thwarted belongingness and capability for suicide was not predictive of future suicide attempts, but perceived burdensomeness showed a significant main effect (*z* = 3.49, *P* < 0.01; OR = 2.34, 95% CI 1.59–3.58) and moderate performance in screening for future suicide attempts (area under the curve AUC = 0.729, *P* < 0.01).

**Conclusions:**

The results challenge the theoretical validity of the IPTS and its clinical utility – at least within the methodological limitations of the current study. Yet, findings underscore the importance of perceived burdensomeness in understanding suicidal ideation and behaviour.

Suicidal thoughts and behaviours are major public health problems that have devastating impacts on individuals and families: the number of suicides worldwide each year is equivalent to one death by suicide every 40 s, and many more attempt suicide.^1^ Therefore, understanding suicide and development of methods to predict and prevent it are of global importance. Determination of risk and protective factors is a critical component in this endeavour: knowledge about suicide risk factors is essential for developing theoretical models, risk assessment strategies and effective treatments. However, in a recent meta-analysis including 365 longitudinal studies Franklin and colleagues^2^ found that existing risk factors are only weak and inaccurate predictors of suicidal behaviour. Analyses also revealed that predictive ability has not improved over the past 50 years. Their conclusion that existing factors rarely correctly identify people who are at risk of suicidal behaviour is supported by an array of further meta-analyses focusing on well-established risk factors such as depression, hopelessness,^3^ sociodemographic factors^4^ and externalising psychopathology.^5^

However, none of these meta-analyses takes into account recent theoretical developments in the field of suicidology. In recent years, a number of theories have been presented that not only try to explain how thoughts of suicide arise but also try to delineate factors that predict the transition from suicidal ideation to suicidal behaviour. The first and most popular of these so-called ‘ideation-to-action’ models is the interpersonal theory of suicide (IPTS) by Joiner^6^ (also known as the interpersonal-psychological theory of suicide and the interpersonal theory of suicidal behaviour). According to the IPTS, two proximal and causal risk factors must be present for someone to desire suicide: perceived burdensomeness and thwarted belongingness. Perceived burdensomeness refers to the view that one's existence is a burden on family members, friends and/or society (‘My family would be better off if I were gone’). Thwarted belongingness refers to the experience that one is alienated from others, not an integral part of a family, circle of friends or another valued group (‘I don't belong to anyone’). The theory suggests that each of these cognitive–affective states is sufficient to elicit passive suicidal ideation (i.e. the wish to die); yet, it is their interaction – combined with the perception that these states are stable and unchanging – that will cause an active desire for suicide (i.e. serious thoughts of taking one's own life). Most importantly, the theory posits that suicidal behaviour occurs only when suicidal ideation is present within the context of acquired capability. Acquired capability for suicide is supposed to comprise two dimensions: elevated pain tolerance and fearlessness about death and dying. A person has to have both to be able to act on a suicidal wish. Joiner^6^ proposed that the most direct route to acquire capability for suicide is by engaging in self-injurious behaviour (e.g. non-suicidal self-injury, suicide attempts); however, one can also become less fearful of pain, injury and death through other painful and provocative events (e.g. combat exposure, childhood abuse). In view of the fact that dispositional factors contribute to fearlessness and pain tolerance, the term ‘acquired capability for suicide’ has now been replaced by the comprehensive term ‘capability for suicide’.^7^

The IPTS is considered one of the most important theories of suicide^8^ and its main assumptions are supported by a large number of studies.^7,9^ However, only a few studies have investigated and supported the role of the three-way interaction of thwarted belongingness, perceived burdensomeness and capability for suicide in suicidal behaviour^10,11^ – and even less did so in a prospective study design. Yet, the theory asserts that its three primary constructs together predict *future* risk of a suicide attempt.^6^ Therefore the main assumptions of the IPTS can only be tested rigorously in a prospective study design. Nonetheless, available prospective studies focus nearly exclusively on suicidal ideation (e.g.^12–17^). Only two studies^11,18^ focused on suicide attempts. However, both made use of proxy measures to assess capability for suicide (i.e. lifetime suicide attempts and non-suicidal self-injury), which does not allow adequate rigorous testing of the theory: first, capability for suicide is not reducible to one single indicator and second, taking self-injurious behaviour as a proxy measure of capability for suicide runs the risk of mixing cause and consequence of capability for suicide.

In summary, no research team has simultaneously examined perceived burdensomeness, thwarted belongingness and capability for suicide in a single study that: (a) used validated multi-item measures of all constructs; (b) implemented in a longitudinal study design; and (c) focused on a sample of high-risk psychiatric in-patients. Against this background, the aim of this study was to test the hypothesis that the three-way interaction of thwarted belongingness, perceived burdensomeness and capability for suicide (including lowered fear of death and elevated pain tolerance) predicts future suicide attempts in a sample of high-risk psychiatric in-patients admitted to a hospital because of either a recent suicide attempt or serious suicidal ideation.

## Method

### Participants

An *a priori* power analysis using GPower 3.1 for Windows was conducted with *α* = 0.05, *β* = 0.95 and *f* = 0.15, which resulted in a minimal sample size of *n* = 195. We expected a drop-out rate of 25–30% (see^19^), so that *n* = 260 were to be included at baseline. A total of 531 patients were approached, of whom 72 did not fulfil the inclusion criteria and 151 refused to participate. The remaining 308 patients fulfilled inclusion criteria and agreed to participate (53.6% female (*n* = 165); mean age 36.82 years, s.d. = 14.30, range 18–81). Participants were admitted to a psychiatric hospital because of severe suicidal ideation (*n* = 145, 47.1%) or a suicide attempt within 2 weeks of admission. The short version of the Diagnostisches Interview bei Psychischen Störungen (Diagnostic Interview for Mental Disorders) (DIPS)^20^ was used to assess patients’ diagnoses. The most common main diagnoses (including comorbidity) according to ICD-10^21^ were affective disorders (F3: *n* = 235; 76.3%), neurotic, stress-related and somatoform disorders (F4: *n* = 110, 35.71%) and personality disorders (F6, *n* = 76, 24.68%).

Prior to assessments, participants were informed about the purpose of the study, the voluntary nature of their participation, as well as data storage and security. They gave written informed consent before participating. The authors assert that all procedures contributing to this work comply with the ethical standards of the relevant national and institutional committees on human experimentation and with the Helsinki Declaration of 1975, as revised in 2008. All procedures involving human participants were approved by the responsible ethics committees (EK 310/13, Medical Faculty of RWTH Aachen University; 4909-14, Medical Faculty of the Ruhr-University Bochum; 042-14-27012014, Medical Faculty of the University of Leipzig).

### Procedure

Participants were recruited in 13 different German hospitals. Patients were interviewed after admission to a psychiatric ward because of attempted suicide or severe suicidal ideation. Exclusion criteria were: age below 18 years, insufficient knowledge of the German language, acute psychotic symptoms, and cognitive impairment or dementia. Participants were reassessed after 3 (T_1_), 9 (T_2_), and 12 (T_3_) months. Data collection took place between September 2016 and February 2018. If participants were not reached for follow-up assessment, up to 10 additional phone calls (including calls to the treating psychiatrist, general practitioner or family members) and up to three attempts via mail or email were made. If there was still no possibility to get in contact with the participant for the respective assessment, new contact attempts were undertaken at the next assessment. Participants were paid €20 for each completed assessment, i.e. €80 in total. The first assessment took place while participants were still in hospital, the T_1_ and T_2_ assessments were conducted via telephone and questionnaires were sent by mail. The T_3_ assessment was conducted face to face at the respective research site in Aachen, Bochum or Leipzig, or, if participants were still or again in hospital, in a separate room in the psychiatric ward. In some cases, if both options were not possible, participants were visited at home. If participants had moved to a place too far away to allow for a face-to-face assessment, interviews were conducted via telephone and questionnaires were sent by mail. In case of an acute suicidal crisis during the assessments, the interviewer would have referred the respective participant to a psychiatric ward. However, such acute crises did not occur during the assessments.

### Instruments

#### Self-Injurious Thoughts and Behaviors Interview (SITBI)

The SITBI^22,23^ is a structured interview that assesses the presence, frequency and characteristics of a wide range of suicidal and self-injurious thoughts and behaviours. Within the current analysis, it was used to assess (a) the lifetime history of suicide attempts (item 40 of the German version of the SITBI: ‘How many suicide attempts have you made in your lifetime?’) and (b) the number of suicide attempts within the follow-up intervals (item 40 was adapted accordingly). If a participant did not attend T_1_ or T_2_ assessments but was reachable at the T_3_ assessment, the SITBI was adopted to ask for the number of suicide attempts since the last assessment. If no follow-up data on suicide reattempt were available, the participant dropped out of analysis. Good interrater and test–retest reliability as well as good convergent validity have been shown for the German version of the SITBI.^23^

#### German Capability for Suicide Questionnaire (GCSQ)

The GCSQ^24,25^ consists of 11 items, 5 of which assess fearlessness about death (e.g. ‘The prospect of my own death is frightening me’) and 5 assess pain tolerance (e.g. ‘If I feel pain, I bite my teeth together and just move on’), the two facets of capability for suicide according to Joiner's theory. One item asks for the participants’ perceived capability for suicide. All items are rated from 1 (fully agree) to 5 (do not agree at all). Higher overall mean scores indicate a higher level of capability for suicide. Construct validity and internal consistency (Cronbach's *α* = 0.79–0.90) were good in prior studies.^25^ Internal consistency in the present sample was *α* = 0.79 (s.e. = 0.02; 95% CI 0.76–0.82).

#### Interpersonal Needs Questionnaire (INQ)

The INQ^26^ assesses perceived burdensomeness with six items (e.g. ‘These days, the people in my life would be happier without me’) and thwarted belongingness with nine items (e.g. ‘These days, I feel disconnected from other people’). All items are to be answered on a seven-point Likert scale ranging from 1 (not at all true for me) to 7 (very true for me), with higher scores indicating higher levels of thwarted interpersonal needs. The German version of the INQ showed very good to excellent internal consistency (α = 0.89 for thwarted belongingness and 0.94 for perceived burdensomeness).^27^ Internal consistency in the present sample was *α* = 0.92 (s.e. = 0.01; 95% CI 0.91–0.94) for perceived burdensomeness and *α* = 0.83 (s.e. = 0.02; 95% CI 0.79–0.85) for thwarted belongingness.

#### Rasch-based Depression Screening (DESC-I)

The DESC-I^28,29^ comprises 10 items referring to the past 2 weeks, which are answered on a five-point Likert scale ranging from 0 (never) to 4 (always) (e.g. ‘How many times in the past two weeks have you felt desperate?’). Higher scores indicate higher levels of depression. The DESC has good psychometric properties with excellent internal consistency in prior studies (Cronbach's α ≥ 0.92).^28,30^ Internal consistency in the present sample was Cronbach's *α* = 0.92 (s.e. = 0.01; 95% CI 0.91–0.94).

#### Beck Hopelessness Scale (BHS)

The BHS^31,32^ includes 20 true/false items that assess pessimistic and hopeless cognitions (e.g. ‘I might as well give up, because there is nothing I can do to improve the situation’). Higher scores indicate higher levels of hopelessness. Validity and reliability of the German version of the BHS has been shown in prior studies.^32^ Internal consistency in the current study was Cronbach's *α* = 0.92 (s.e. = 0.001; 95% CI 0.91–0.95).

#### Beck Scale for Suicide Ideation (BSS)

The BSS^33,34^ is a self-report questionnaire assessing suicidal ideation. It comprises 21 statement groups, each consisting of three response options with increasing severity (ranging from 0 to 2) referring to the past 7 days (e.g. ‘I have no wish to die/a weak wish to die/a moderate to strong wish to die’). Items 1–5 of the BSS can be used as a screening tool to assess suicidal ideation, and the total scale (19 items) captures the severity of suicidal ideation. There are two additional items,^20,21^ which assess the number of past suicide attempts and their severity. The sum score of the BSS (items 1–19) therefore ranges from 0 to 38, with higher values indicating increased suicide risk. Studies on the factorial structure of the BSS revealed inconsistent results; therefore, the use of the total score is common practice.^35^ The internal consistency in our sample was good for the total score (Cronbach's *α* = 0.87; s.e. = 0.001; 95% CI 0.85–0.89) and comparable with the psychometric properties (*α* = 0.88) reported by Kliem & Braehler.^33^

### Statistical analysis

Two participants reported lifetime number of suicide attempts ≥100, which was considered implausible, so these individuals were excluded from the present analyses. There is no consensus on dealing with missing values in suicidology; in accordance with Wetherall et al,^36^ we opted for a tolerance of 25% missing values per scale. Therefore, an individual's data for the respective scale was excluded if they had not responded to at least 75% of the items. After applying the criterion, values for a maximum of two participants per scale had to be excluded. First, means and standard deviations (s.d.) were calculated for all variables and all participants. Those participants with full information on suicide reattempt within the 12-month follow-up were compared with those participants without full follow-up information on all study variables (drop-out analysis).

In the next step, we analysed whether participants with a suicide attempt within the 12-month follow-up interval differed from those without a suicide attempt with regard to perceived burdensomeness, thwarted belongingness, capability for suicide, hopelessness, depression, number of lifetime suicide attempts and demographic variables (age, gender, family status) using *t*-tests for independent groups, regression analyses and *χ*^2^-tests.

To examine the IPTS assumption that the interaction between perceived burdensomeness, thwarted belongingness and capability for suicide at baseline predicts suicide attempt within the 12-month follow-up, logistic regression analyses were conducted. Perceived burdensomeness, thwarted belongingness and capability for suicide were entered in the first step; in the second step, the two-way interactions between them were entered; the three-way interaction of perceived burdensomeness, thwarted belongingness and capability for suicide was entered in the fourth step. Significant predictors were then entered into a receiver operating curve (ROC) analysis^37^ to evaluate their overall performance to screen for risk of future suicide attempt. The area under the curve (AUC) directly represents the accuracy of the instrument in screening for depression. Swets^38^ suggests heuristically interpreting AUC values as small (0.5 < AUC < 0.7), moderate (0.7 < AUC < 0.9), or high (0.9 < AUC < 1).

In all regression analyses, centring and *z*-standardization of predictors was performed to facilitate the interpretation of the regression weights. Following the argumentation by Rogers et al,^39^ we did not control for depression. Predictors were checked for multicollinearity (variance inflation factor VIF < 1.3 for all predictors). Analyses were conducted with R, version 3.6.1 for Windows, using packages haven, psych, mbess and lmtest. The ROC analysis was conducted with SPSS, version 25 for Windows.

## Results

### Drop-out analysis

Out of the entire study sample of 308 participants, 5 refused to participate in the T_1_ assessment and 84 were not reached. Two participants died by suicide between T_0_ and T_1_, and an additional 2 had to be excluded because of acute psychotic symptoms. Nine individuals refused to participate in the T_2_ assessment, 94 were not reached and 1 died of natural causes. Eight individuals refused to participate in the T_3_ assessment and 116 were not reached. In total, more than half of the participants initially included in the study (*n* = 175; 56.82%) provided full information on suicide reattempts within the entire 12-month follow-up interval and were thus included in the analysis. Forty-three (24.6%) of these individuals reported at least one suicide attempt within the 12-month follow-up interval. There were 24 participants reporting at least one suicide attempt within 6 months (T_1_), 16 in the period between 6 and 9 months after baseline (T_2_) and 17 in the period between 9 and 12 months after baseline (T_3_) (including individuals reporting repeated attempts). As shown in supplementary Tables 1 and 2, available at https://doi.org/10.1192/bjo.2020.93, participants with full follow-up information on suicide reattempts did not differ from those without full follow-up information (considered as having dropped out in the current analysis: *n* = 133; 43.18%) on any study variable, age, gender, family status or diagnosis.

### Differences between participants with and without a suicide attempt within the 12-month follow-up interval

Table 1 shows diagnoses, gender and family status for the 175 participants with full information on suicide reattempts within the entire 12-month follow-up interval.

**Table 1:** Differences in diagnoses, gender and family status for participants with a suicide attempt and those without a suicide attempt within the 12-month follow-up interval

	Participants with no suicide attempt (*n* = 132)^a^		Participants with a suicide attempt (*n* = 43)^b^		χ^2^	d.f.	*P*
	*n*	%	*n*	%			
ICD-10 diagnoses							
F0	1	0.76	0	0.00	0.00	1	1.00
F1	17	12.88	12	27.91	4.39	1	0.04
F2	1	0.76	0	0.00	0.00	1	1.00
F3	106	80.30	31	72.09	0.74	1	0.39
F4	42	31.82	17	39.53	0.61	1	0.43
F5	5	3.79	5	11.63	2.44	1	0.12
F6	20	15.15	19	44.19	14.48	1	0.00
F7	0	0.00	0	0.00	–	–	–
F8	1	0.76	0	0.00	0.00	1	1.00
F9	2	1.52	1	2.33	0.00	1	1.00
Gender					3.09	2	0.21
Female	75	56.8	24	55.8			
Male	57	43.2	18	41.9			
Diverse	0	0	1	2.3			
Family status					4.87	4	0.30
Single	55	41.7	14	32.6			
Partnership	25	18.9	9	20.9			
Married	27	20.5	5	11.6			
Divorced	20	15.2	11	25.6			
Widowed	1	0.8	1	2.3			

F0: Organic, including symptomatic, mental disorders; F1: Mental and behavioural disorders due to psychoactive substance use; F2: Schizophrenia, schizotypal and delusional disorders; F3: affective disorders; F4: Neurotic, stress-related and somatoform disorders; F5: Behavioural syndromes associated with physiological disturbances and physical factors; F6: Disorders of adult personality and behaviour; F7: Mental retardation; F8: Disorders of psychological development; F9: Behavioural and emotional disorders with onset usually occurring in childhood and adolescence.

a. 75.43% of the total sample with full information on suicide reattempts within the entire 12-month follow-up interval.

b. 24.57% of the total sample with full information on suicide reattempts within the entire 12-month follow-up interval.

Table 2 shows that participants with a suicide attempt within the 12-month follow-up interval had significantly higher baseline scores for depression, perceived burdensomeness, thwarted belongingness, hopelessness, suicidal ideation and number of lifetime suicide attempts. However, out of the four indicators applied to measure capability for suicide (GCSQ total score, the fearlessness about death and pain tolerance subscales, and the perceived capability for suicide item), only the perceived capability item differed significantly between the two groups, with higher mean scores in the attempter group than in the non-attempter group.

**Table 2:** Differences between patients with a suicide attempt and those without a suicide attempt within the 12-month follow-up interval

	Participants with no suicide attempts (*n* = 132)		Participants with a suicide attempt (*n* = 43)		*t*-test			Logistic regression^a^					
	Mean	s.d.	Mean	s.d.	*t*	d.f.	*P*	*B*	s.e.	*z*	*P*	OR	95% CI^b^
Age, years	38.76	15.16	34.83	12.91	1.63	77.52	0.11	−0.28	0.19	−1.49	0.14	0.76	0.55–1.02
DESC-I depression score	24.79	8.59	30.36	6.04	−4.60	95.72	<0.01	0.88	0.25	3.56	<0.01	2.42	1.64–3.72
INQ perceived burdensomeness score	3.14	1.73	4.59	1.65	−4.85	69.88	<0.01	0.85	0.20	4.22	<0.01	2.33	1.69–3.28
INQ thwarted belongingness score	3.87	1.33	4.62	1.25	−3.29	70.89	<0.01	0.60	0.20	3.02	<0.01	1.81	1.32–2.53
GCSQ capability for suicide total score	37.49	8.94	38.98	9.87	−0.86	61.93	0.39	0.16	0.18	0.91	0.36	1.18	0.88–1.59
GCSQ fearlessness about death score	16.11	6.19	16.04	6.76	0.06	62.46	0.95	−0.01	0.18	−0.06	0.95	0.99	0.74–1.33
GCSQ pain tolerance score	17.42	4.40	18.46	5.20	−1.15	59.02	0.25	0.23	0.19	1.25	0.21	1.26	0.93–1.72
GCSQ perceived capability for suicide score	3.95	1.21	4.45	0.93	−2.73	82.88	0.01	0.52	0.23	2.29	0.02	1.69	1.19–2.53
BHS hopelessness score	12.27	5.56	14.54	4.69	−2.57	79.03	0.01	0.46	0.20	2.29	0.02	1.59	1.15–2.24
BSS suicide ideation score	13.14	9.50	18.58	8.67	−3.42	72.82	<0.01	0.61	0.20	3.09	<0.01	1.85	1.34–2.60
Lifetime suicide attempts, *n*	1.37	2.18	3.94	3.75	−4.26	51.60	<0.01	0.30	0.07	4.15	<0.01	1.35	1.21–1.54

GCSQ, German Capability for Suicide Questionnaire; INQ, Interpersonal Needs Questionnaire; DESC-I, Rasch-based Depression Screening; BHS, Beck Hopelessness Scale; BSS; Beck Scale for Suicide Ideation; OR, odds ratio.

a. Regression analysis predicted group membership (patients with versus without a suicide attempt).

b. 95% CI: lower and upper level of 95% confidence interval.

Moreover, a significantly larger fraction of the participants with a suicide attempt within the 12-month follow-up interval had ICD-10 F1 or F6 diagnoses (Table 1).

### Multifactorial prediction of suicide attempts

Results of a hierarchical linear regression analysis entering perceived burdensomeness, thwarted belongingness and capability for suicide in the first step, two-way interactions in the second step and the three-way interaction in the third step are summarised in Table 3. Perceived burdensomeness showed a significant main effect on suicide attempts within the 12-month interval, which remained significant even after entering all interaction terms into the model. No other predictor was significantly related to the dependent variable. Perceived burdensomeness at baseline was entered into a ROC analysis. Results revealed a moderate performance of perceived burdensomeness in screening future suicide attempt risk (AUC = 0.729; 95% CI 0.645–0.814; *P* < 0.01).

**Table 3:** Logistic regression analyses predicting suicide attempt within the 12-month follow-up by baseline variables of the interpersonal theory of suicide

	Step 1						Step 2						Step 3					
	*B*	s.e.	*z*	*P*	OR	95% CI^a^	*B*	s.e.	*z*	*P*	OR	95% CI^a^	*B*	s.e.	*z*	*P*	OR	95% CI^a^
Intercept	−1.37	0.21	−6.47	<0.01	0.26	0.18–0.36	−1.34	0.22	−6.04	<0.01	0.26	0.18–0.37	−1.37	0.23	−5.94	<0.01	0.25	0.17–0.36
Perceived burdensomeness	0.72	0.21	3.38	<0.01	2.06	1.46–2.96	0.78	0.22	3.47	<0.01	2.18	1.52–3.20	0.85	0.24	3.49	<0.01	2.34	1.59–3.58
Thwarted belongingness	0.33	0.22	1.53	0.13	1.39	0.98–1.99	0.37	0.24	1.52	0.13	1.45	0.98–2.18	0.40	0.25	1.59	0.11	1.49	0.99–2.29
Capability for suicide	0.10	0.20	0.53	0.60	1.11	0.80–1.54	0.18	0.22	0.81	0.42	1.20	0.83–1.75	0.12	0.23	0.53	0.60	1.13	0.77–1.68
Perceived burdensomeness × thwarted belongingness							−0.16	0.23	−0.69	0.49	0.86	0.58–1.23	−0.29	0.26	−1.12	0.26	0.75	0.48–1.13
Thwarted belongingness × capability for suicide							0.12	0.23	0.54	0.59	1.13	0.78–1.67	0.03	0.24	0.12	0.91	1.03	0.70–1.53
Perceived burdensomeness × capability for suicide							−0.28	0.22	−1.31	0.19	0.75	0.52–1.07	−0.41	0.24	−1.69	0.09	0.67	0.44–0.97
Perceived burdensomeness × thwarted belongingness × capability for suicide													0.35	0.23	1.52	0.13	1.42	0.98–2.08
Model (Wald)	*χ^2^* = 19.45; *P* < 0.01						*χ^2^* = 19.41; *P* < 0.01						*χ^2^* = 19.12; *P* < 0.01					

OR, odds ratio.

a. 95% CI: lower and upper level of 95% confidence interval.

## Discussion

The current study examined a central assumption of the IPTS regarding the prediction of future suicide attempts in a high-risk sample of adults admitted to a psychiatric hospital because of a suicide attempt or severe suicidal ideation within 12 months after the initial assessment. The primary finding was that, contrary to expectation, the interaction between thwarted belongingness, perceived burdensomeness and capability for suicide did not predict suicide attempt in the 12-month follow-up-period. Although unexpected and contrary to the theoretical assumptions, this finding is consistent with retrospective studies in in-patient populations^40,41^ as well as two prospective studies in adolescent populations,^11,18^ neither of which found support for this main assumption of the IPTS. To our knowledge, Joiner et al^10^ conducted the only study that found the three-way interaction to be predictive of suicide attempts in an in-patient sample. However, that study – and also the prospective studies by Czyz et al^18^ and King et al^11^ – made use of proxy measures to assess capability for suicide and focused on past instead of future suicide attempts. Unlike all previous studies, the current study made use both of validated measures originally developed to assess the theory's main constructs and a prospective study design.

Overall, the results of the current study question the relative importance and utility of the three IPTS constructs in explaining suicidal behaviour – at least with regard to in-patient samples. However, from a clinical perspective, knowledge about risk factors is especially important in dealing with high-risk patients. Therefore, the results challenge the clinical utility of employing the IPTS to predict suicide risk (see^7^). Still, in line with previous research,^7,9^ the current results highlight the importance of perceived burdensomeness in understanding suicidal ideation and behaviour. Perceived burdensomeness showed a significant main effect on suicide attempt within the 12-month interval, which remained significant even after entering all interaction terms into the regression model. However, the direct pathway between perceived burdensomeness and suicide attempts is not part of the IPTS. According to the IPTS, one would expect a main effect of perceived burdensomeness only on passive suicidal ideation (i.e. a wish to die), but not on active suicidal ideation or on suicidal behaviour. Furthermore, current effect sizes point to the fact that perceived burdensomeness seems to be a no better predictor of suicide risk than other (traditional) risk factors (e.g. depression, hopelessness, suicidal ideation^42^). Nonetheless, perceived burdensomeness could be a relevant target for treatment.^43^

### Implications

Taken together, the present results could be interpreted as being in line with recent claims that accurate prediction of suicidal behaviour will likely require a complex combination of a large number of risk and protective factors.^42^ Moreover, it is likely that there are many different paths to suicidal behaviour. This equifinality means that a single one-size-fits-all algorithm might be too simplistic to allow sufficient understanding of suicidal ideation and suicidal behaviour in different subgroups or cultures.^44^ On a theoretical level, the integrated motivational–volitional (IMV) model of suicidal behaviour^45^ might offer a framework within which the complex interaction of various risk factors is mapped. The central constructs of the IPTS – perceived burdensomeness, thwarted belongingness, capability for suicide – are taken into account within the IMV model, but without having greater weight than other variables. There is one prospective study offering initial support for a core assumption of the IMV model.^46^ Nonetheless, the relevance of the complex interplay of risk factors outlined in the model has yet to be tested in prospective studies. On an empirical level, recent developments in using machine learning approaches to predict suicidal ideation and suicide attempts (e.g.^47,48^) open a completely new way to consider a large number of predictors and their interaction simultaneously. A shift away from a focus on risk factors to a focus on risk algorithms has the potential to advance the field; yet research in this area is inconclusive^49^ and the prediction of suicidal behaviour is likely to stay an approximation.^50^

### Limitations and strengths

The current study, although using validated measures, a large sample and a prospective study design, has some limitations. First, 43.18% of participants enrolled in this prospective study were lost to follow-up, which is more than expected and could have negatively affected the power to detect a significant effect. Although non-completers did not differ from completers, a systematic response bias cannot be fully ruled out. Second, the IPTS was designed to explain the occurrence of lethal or near-lethal suicidal behaviour. The current study's focus on suicide attempts of any degree of lethality might therefore be an imprecise test of the theory's assumptions. Future studies, using larger samples and death by suicide rather than suicide attempts as an outcome are needed (but very laborious and costly) to ultimately test the theory. Third, the theory proposes that perceived burdensomeness, thwarted belongingness and capability for suicide are proximal risk factors. The prediction of suicide attempts over a period of 12 months may therefore not do justice to the assumptions of the theory – especially since all three constructs are subject to significant fluctuations over time.^13,51^ Nonetheless, the current study has important methodological advantages in contrast to prior work, such as use of validated measures originally developed to assess the theory’s main constructs and a prospective study design.

It has to be noted that the present study was not pre-registered. Since our study was not an interventional trial, pre-registration was not mandatory. Generally, pre-registration of studies and open science foster research transparency and aim at preventing research misconduct.^52,53^ Since our study was funded by the German Research Foundation (Deutsche Forschungsgemeinschaft) a detailed grant application was submitted in June 2015, thus prior to starting the project. To enhance transparency of our research, the section of this grant proposal on study hypotheses and any information on study methods and data analyses can be obtained from the corresponding author on request. A summary of the project can be found at https://gepris.dfg.de/gepris/projekt/288645884?language=en.

Future studies should seek to address the limitations mentioned above and should try to take into account the complexity of suicidal ideation and suicidal behaviour to a greater extent.

## Data Availability

The script for the analyses as reported in Tables 1–3 and online supplementary Tables 1 and 2 is provided as an additional online supplementary file. The data can be obtained from the corresponding author on request. The agreement to the general anonymised provision of the data as an online resource was unfortunately not part of the written informed consent that the participants signed before enrolment.

## Supplementary material

For supplementary material accompanying this paper visit http://dx.doi.org/10.1192/bjo.2020.93. click here to view supplementary material
