# Supplementary material for: Interpersonal theory of suicide: prospective examination
Source: BJPsych Open. 2020 Sep 22;6(5):e113. doi: 10.1192/bjo.2020.93 (PMC7576651; doi:10.1192/bjo.2020.93)
Supplement: Supplementary file 1 [file S2056472420000939sup001.zip › S2056472420000939sup010.docx]

**Online supplementary material**

Table A: comparison between patients with full follow-up information on suicide reattempts and those without full information

|  | **only T0 data** | | **T0 and 12 months follow up** | |  |  |  |
| --- | --- | --- | --- | --- | --- | --- | --- |
|  | **M** | **SD** | **M** | **SD** | **t** | **df** | **p** |
| **age** | 35.73 | 13.77 | 37.83 | 14.71 | -1.27 | 284.77 | 0.21 |
| **depression** | 27.38 | 8.26 | 26.13 | 8.38 | 1.27 | 267.46 | 0.21 |
| **PB** | 3.66 | 1.94 | 3.48 | 1.82 | 0.81 | 254.47 | 0.42 |
| **TB** | 3.87 | 1.3 | 4.05 | 1.35 | -1.13 | 267.74 | 0.26 |
| **CS** | 37.91 | 8 | 37.85 | 9.17 | 0.06 | 283.35 | 0.95 |
| **hopelessness** | 12.61 | 5.74 | 12.82 | 5.44 | -0.31 | 259.13 | 0.76 |
| **suicide ideation** | 15.08 | 9.47 | 14.45 | 9.57 | 0.56 | 269.01 | 0.58 |
| **# suicide attempts lifetime** | 3.01 | 5.62 | 2.01 | 2.87 | 1.85 | 173.87 | 0.07 |

note: PB: perceived burdensomeness. TB: thwarted belongingness. CS: capability for suicide (total),
