# Supplementary material for: Interpersonal theory of suicide: prospective examination
Source: BJPsych Open. 2020 Sep 22;6(5):e113. doi: 10.1192/bjo.2020.93 (PMC7576651; doi:10.1192/bjo.2020.93)
Supplement: Supplementary file 1 [file S2056472420000939sup001.zip › S2056472420000939sup011.docx]

**Online supplementary material**

Table B: Diagnoses, gender, and family status for patients with full follow-up information on suicide reattempts and those without full information

|  |  | **all participants** | | **only T0 data** | |  |  |  | **T0 and 12 months follow up** | |  |  |  | ***χ^2^*** | ***df*** | ***p*** |
| --- | --- | --- | --- | --- | --- | --- | --- | --- | --- | --- | --- | --- | --- | --- | --- | --- |
|  |  | **N** | **%** | **N** | **%** |  |  |  | **N** | **%** |  |  |  |  |  |  |
| **sample size** |  | 308 |  | 133 | 43.18 |  |  |  | 175 | 56.82 |  |  |  |  |  |  |
| **diagnoses** |  |  |  |  |  |  |  |  |  |  |  |  |  |  |  |  |
| (ICD-10) | **F0** | 4 | 1.30 | 3 | 2.26 |  |  |  | 1 | 0.57 |  |  |  | 0.65 | 1 | 0.42 |
|  | **F1** | 61 | 19.81 | 31 | 23.31 |  |  |  | 30 | 17.14 |  |  |  | 1.63 | 1 | 0.20 |
|  | **F2** | 8 | 2.60 | 6 | 4.51 |  |  |  | 2 | 1.14 |  |  |  | 2.27 | 1 | 0.13 |
|  | **F3** | 235 | 76.30 | 97 | 72.93 |  |  |  | 138 | 78.86 |  |  |  | 0.67 | 1 | 0.41 |
|  | **F4** | 110 | 35.71 | 50 | 37.59 |  |  |  | 60 | 34.29 |  |  |  | 0.35 | 1 | 0.55 |
|  | **F5** | 16 | 5.19 | 6 | 4.51 |  |  |  | 10 | 5.71 |  |  |  | 0.03 | 1 | 0.86 |
|  | **F6** | 76 | 24.68 | 36 | 27.07 |  |  |  | 40 | 22.86 |  |  |  | 0.65 | 1 | 0.42 |
|  | **F7** | 0 | 0.00 | 0 | 0.00 |  |  |  | 0 | 0.00 |  |  |  | -- | -- | -- |
|  | **F8** | 2 | 0.65 | 1 | 0.75 |  |  |  | 1 | 0.57 |  |  |  | 0.00 | 1 | 1.00 |
|  | **F9** | 4 | 1.30 | 1 | 0.75 |  |  |  | 3 | 1.71 |  |  |  | 0.05 | 1 | 0.83 |
|  |  |  |  |  |  |  |  |  |  |  |  |  |  |  |  |  |
|  |  |  |  | **female** | **male** | **diverse** |  |  | **female** | **male** | **diverse** |  |  |  |  |  |
| **gender** |  |  |  | 66 | 67 | 0 |  |  | 99 | 75 | 1 |  |  | 2.34 | 2 | 0.31 |
|  |  |  |  | **single** | **partnership** | **married** | **divorced** | **widowed** | **single** | **partnership** | **married** | **divorced** | **widowed** |  |  |  |
| **family status** |  |  |  | 55 | 21 | 21 | 21 | 6 | 69 | 34 | 32 | 31 | 2 | 4.33 | 4 | 0.36 |
